# Effects of Selected Parameters on the Bonding Quality and Temperature Evolution Inside Plywood During Pressing

**DOI:** 10.3390/polym12051035

**Published:** 2020-05-02

**Authors:** Pavlo Bekhta, Ján Sedliačik, Nataliya Bekhta

**Affiliations:** 1 Institute of Woodworking and Computer Technologies and Design, Ukrainian National Forestry University, 79057 Lviv, Ukraine; n.bekhta@nltu.edu.ua; 2 Faculty of Wood Sciences and Technology, Technical University in Zvolen, 960 01 Zvolen, Slovakia; jan.sedliacik@tuzvo.sk

**Keywords:** plywood, densification, core layer temperature, bonding quality, hot pressing, veneer stack heating

## Abstract

This research optimizes the process of plywood production to determine its effectiveness in reducing energy and adhesive consumption for more efficient production with the required quality. The influence of selected parameters including veneer treatment (non-densified and densified), plywood structure, temperature, time and pressure of pressing, on the bonding quality and temperature evolution within the veneer stacks during hot pressing was investigated. Rotary-cut, non-densified and densified birch veneers and phenol formaldehyde (PF) adhesive were used to manufacture plywood samples. The effect of pressure and time of pressing on bonding quality of the plywood was determined. Bonding quality was evaluated by determining the shear strength of the plywood samples. The temperature evolution inside the veneer stacks was measured for birch veneers for different pressing temperatures and pressures for different numbers of veneer layers. The heating rate of the veneer stacks increased as the pressing temperature increased and decreased markedly with an increasing number of veneer layers. At a high pressing pressure, the heating rate of the densified veneer stacks was faster than that of non-densified veneers at the same pressure. The use of densified veneers for the production of plywood can lead to a shorter pressing time (17–50% reduction), lower glue consumption (33.3% reduction) and a lower pressing pressure (22.2% reduction) without negatively impacting the bonding strength of the plywood.

## 1. Introduction

The structure and properties of plywood are formed in the hot-pressing process. Hot pressing is one of the most important operations in the production of plywood, which has an impact on the properties of plywood. This operation is also important from an economic point of view. The hot press step determines the performance of the pressing line and defines the capacity of the factory. Hot pressing is one of the most energy-consuming processes in plywood manufacturing after the veneer-drying process. Hot-pressing parameters such as pressing time, pressure and temperature are key factors that directly affect the properties of plywood panels [1,2,3]. Despite that plywood was the first created wood-based composite, few individual studies have been conducted to investigate the effect of process variables on hot-pressing of plywood [1,3,4,5,6,7,8]. The temperature evolution within the panel during hot-pressing is important for the chemical and physical processes that contribute to the properties of the panel. The temperature evolution within the panel depends on wood species, their density and moisture content, compression of the veneers, glue spreading rate, and the pressing temperature, pressure and time. The interactions among these parameters are however, complicated and unclear [9].

Pressing pressure depends on wood species, physical properties of the wood, characteristics of the veneer surface and on the type of adhesive, its viscosity, the pressing temperature, etc. The application of pressure helps the adhesive to wet and penetrate the wood surface by forcing it into the void spaces of the wood [10,11]. However, too high of pressure should be avoided as the adhesive largely squeezes out [12]. Bonding quality is influenced by the amount of adhesive penetration into the wood substrate during the manufacture of wood composites, i.e., plywood [10]. Optimal adhesive penetration is required to repair damaged wood surfaces, and it provides better contact with the inner surface for chemical bonding or blocking and transfers stresses between the laminates [10,12], promoting a more efficient use of adhesive and providing a reliable thickness of the adhesive layer [10].

Pressing pressure and adhesive spread rate are the main factors for determining the thickness of the adhesive layer. The thickness of the adhesive layer should be controlled because it directly affects the strength of the wood composites. Insufficient pressure in the production process results in a thick adhesive layer [13]. As a rule, the thick adhesive layer of many common adhesives is characterized by insufficient strength [11,13]. Pressure should be applied evenly and adequately because wood adhesives based on synthetic resin, such as phenol formaldehyde (PF), are not capable of forming strong bonds in the thick and variable thickness of the adhesive layers due to their low viscosity [12].

In low-density wood species, high pressure causes the adhesive to penetrate so deep into the wood that there is insufficient adhesive to fill the bonding line; this excessive penetration can lead to lower bond strength [11]. By contrast, low pressures can cause a decrease in the shear strength and do not provide close contact between the surfaces, resulting in a poor adhesive layer [13,14].

Thermosetting PF adhesive is typically used in the manufacturing of plywood destined for use in exterior structural applications. To harden the adhesive and form a strong adhesive bond, the temperature inside the veneer stack (in the core layer of the stack) must be greater than 100 °C [14]. The heat from the hot plates of the press should be transferred to the core layer of the stack as quickly as possible. If the heat transfer process to reach a central layer temperature greater than 100 °C is prolonged, then the adhesive layers closer to the plates will be exposed to higher temperatures longer than required, and this can lead to premature hardening of the adhesive in these layers and even destruction of the adhesive. Therefore, for plywood, the total pressing time is determined by the time it takes to reach a sufficient temperature in the inner glue layer to cure the resin (i.e., the temperature at which the resin hardens) [15]. On one hand, to maximize process efficiency, the pressing time should be as short as possible. This is enabled by increasing the pressing temperature. Even a slight increase in the optimum pressing temperature can adversely affect the surface quality and strength of the plywood panels. On the other hand, the pressing should be long enough to allow the glue to harden. Furthermore, shortening the time of hot pressing can effectively reduce energy consumption as well as time necessary for the production of wood composite materials. Therefore, choosing the optimum pressing parameters, i.e., the temperature, pressure and pressing time, is very important, both technologically and economically.

The possibility of reducing hot pressing time has been studied in previous works on some types of wood composite materials, such as laminated veneer lumber (LVL) [15,16], particleboard [17], oriented strand board (OSB) [18] and medium-density fiberboard [19]. However, there are only a few studies in the existing literature regarding the effect of pressing time on the properties of plywood [16,20,21,22,23]. Shortening the duration of plywood pressing can be realized by steam injection [20] or by veneer incising [16,21]. However, the rapid increase in core temperature, typical of steam-injected particleboard, does not occur in steam-injected plywood [20]. Mirski et al. [22] demonstrated that the application of PF resin modified with ethyl malonate enables the production of plywood with good mechanical properties and bond quality in a pressing time shortened by 38% and can reduce the pressing temperature by 20 °C. Li et al. [23] investigated the effects of hot-pressing parameters (temperature, pressure, time and veneer layers) on the shear strength of multi-plywood using modified soy protein adhesives. The authors found that the heating rate of the plywood core layer increased with the increase of hot-pressing temperature and decreased noticeably with an increased number of veneer layers.

Another approach for shortening the heating time and the overall pressing time is the preliminary thermal compression of veneers. In recent years, a number of studies have shown that introducing a veneer preparation process via thermo-mechanical compression prior to applying the adhesive reduces the pressure and time of pressing and also significantly reduces the consumption of adhesive without negatively impacting the bonding quality of the plywood [24,25,26]. Kurowska et al. [27] concluded that veneer densification shortened the total pressing time by 12–25% in comparison to control samples. Bekhta and Salca [8] found that the multilayered plywood made of densified birch veneers with an adhesive spread rate of 150 g/m^2^ was heated faster compared to plywood made of non-densified veneers.

Thus, we hypothesized that thermal compression of the veneers would lead to reaching the curing temperature of the glue in the core layer more rapidly, even at a lower adhesive spread rate, compared with panels made from non-densified veneers, and this will subsequently shorten the pressing time. The purpose of this study was to obtain a better understanding of the heating process of veneer stacks and of the temperature evolution within the plywood panels during hot pressing when using different types of veneer (non-densified and densified), different pressures, time of pressing and pressing temperatures and different numbers of veneer layers. We also investigated the optimal pressing parameters to reduce glue consumption and studied how this will affect the quality of the plywood bonding and increased production efficiency.

## 2. Materials and Methods

### 2.1. Materials

In this study, we used rotary-cut birch (*Betula verrucosa* Ehrh.) veneers (LLC «ODEK» Ukraine) with dimensions of 300 mm × 300 mm and thickness of 1.6 mm and density of 625 kg/m^3^. The average moisture content of the non-densified veneers was 5.7%.

Half of the veneer sheets were densified by the application of heat and pressure between the smooth and carefully cleaned heated plates of an open-system laboratory press at a temperature of 150 °C and a pressure of 2 MPa for 1 min. After densification, the samples were removed from the hot press and allowed to cool to room temperature. The average thickness and moisture content of the densified birch veneers were 1.5 mm and 1.4%, respectively.

The commercial PF adhesive Fenokol 43 EX (Chemko, a. s. Slovakia), with a solid content of 47% (at 105 °С), a viscosity of 278 mPa·s, a gel time of 24 s (at 150 °С), a free phenol content of 0.013%, a free formaldehyde content of 0.032% and a hydrogen ion concentration (рН 11), was used to bond the veneers. The PF resin was used for plywood panel manufacturing without any filler or additive.

### 2.2. Experimental Procedure

In this study, two series of experiments were performed.

During the first series of experiments, the temperature evolution of the adhesive-free plywood samples during hot pressing was measured:

- inside a stack of common non-densified birch veneers at different pressing temperatures (100, 120, 130, 140 and 150 °С);
- inside a stack of either non-densified or densified birch veneers at different pressing pressures (1.0, 1.4 and 1.8 MPa);
- inside a stack of either non-densified or densified birch veneers at different pressing temperatures (100, 130 and 150 °С) and containing different numbers of veneer layers (3, 5 and 7 layers).

In this series of experiments, the measurements of the core layer temperature inside the veneer stacks were carried out without the presence of glue. This was done to determine the real impact of each studied factor on the temperature evolution inside the sample. It was difficult to measure the temperature when adhesive was used because the adhesive in a liquid or solid state would significantly affect the result.

During the second series of experiments, the influence of the pressing parameters, in particular the pressing time (120, 180, 240, 300 and 360 s) and the pressing pressure (1.0, 1.4 and 1.8 MPa), on the properties of PF adhesive-bonded three-layer plywood made from either non-densified or densified birch veneers with reduced adhesive consumption (100 g/m^2^) was studied.

### 2.3. Core Layer Temperature Testing

A thermocouple was placed inside the plywood sample to measure the glue line inner temperature. A 5 mm × 150 mm (width × length) groove was opened in the middle of the core veneer to install the thermocouple. The temperatures were measured with the thermocouple at the center of the central veneer sheet (layer) of the stack and were monitored every 5 s to record the temperature evolution in the panel during pressing. Data collection was initiated when the surface veneer began to receive pressure. When the temperature inside the stack reached the pressing temperature, the test was completed and the data were saved to a computer.

### 2.4. Preparation of Plywood Samples

The three-layer plywood samples from densified veneer were made in an electrically heated hydraulic laboratory press under the following conditions: 150 °C pressing temperature; and different specified values of pressing pressure (1.0, 1.4 and 1.8 MPa) and pressing time (120, 180, 240, 300 and 360 s) and glue spreading (100 and 150 g/m^2^). For comparison, plywood control samples from non-densified veneer were made at the same pressing conditions and pressing pressure of 1.0 and 1.8 MPa. During the last 30 s of the press cycle, the pressure was continuously reduced to 0 MPa. The adhesive was applied onto one side of every uneven ply. The plies were assembled perpendicular to each other (veneer sheets were laid tight/loose) to form plywood of three/five/seven plies. Adhesive was applied onto the veneer surface with a hand roller spreader.

### 2.5. Shear Strength Test

During the experiment, all plywood samples were conditioned prior to testing for 2 weeks at 20 ± 2 °C and 65% ± 5% relative humidity. The panels were cut to extract test samples according to the standard requirements. The shear strength was determined according to methods EN 314-1 [28] and EN 314-2 [29] after pretreatment for their intended use in exterior conditions. For the shear strength test, PF plywood test pieces were immersed for 4 h in boiling water, followed by drying in a ventilated oven for 16 h at 60 ± 3 °C, immersion in boiling water for 4 h, and finally, immersion in cool water at 20 ± 3 °C for at least 1 h. Ten samples were used for each variant shear strength mechanical test.

## 3. Results

During hot pressing, heat is first, transferred from the hot press plates to the outer veneer layers by conduction and then continues to migrate to the middle. The effective porosity in veneer panels was only 0.05–0.5% compared to the total panel voids, which ranged from 50% to 70%. The rate of convection is negligible; thus, heat conduction is dominant [30]. At the same time, upon contact of the surface veneer layers with the hot press plates, the moisture present in these layers turns into steam. The steam migrates to the middle of the panel. However, during hot pressing of veneer panels, it is veneer compression that results in layered and uniform barriers to moisture movement [30]. The interior vapor pressure increases as the steam continues to migrate from the hot surfaces toward the colder middle. In the middle zone of the panel (from which moisture is virtually free to evaporate and, under certain conditions, is contained there in the form of a superheated steam–water mixture), the temperature is continually increasing, approaching the temperature of the press plates [9]. However, the rate of temperature rise in the outer and inner layers of the middle zone of the panel is different [4,9].

### 3.1. Influence of Pressing Temperature on the Heating Rate of the Veneer Stacks

**Figure 1:**
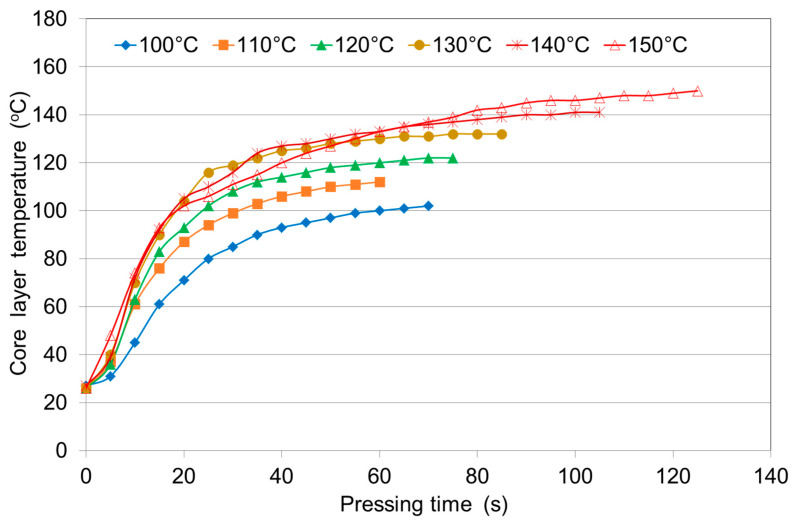
Core layer temperature curves at different pressing temperatures and 1.8 MPa of three-layer plywood made from non-densified birch veneers.

Figure 1 shows how the core layer temperature inside the three-layer birch veneer stack depends on the plywood pressing temperature and Table 1 presents the time required to reach a temperature of 100 °C inside the three-layer panel when applying the different pressing temperatures.

The core layer temperature rose from an initial temperature of 25–30 °C to approximately 100 °C in approximately 20 s when the press was closed, but it took almost 125 s to reach a pressing temperature of 150 °C.

During the gradual heating of the veneer stacks, the wood underwent a temperature change and the water contained within the wood was also altered. At 100 °C, some of the moisture was converted to steam, filling all the spaces within the wood and between the veneer sheets. The rapid heating of the middle of the panel to 100 °C can be explained by the fact that at this temperature liquid water is transformed into steam, which moves from the outer layers to the inner layers, quickly heating the panel to 100 °C.

It is natural that the interior temperature reaches 100 °C most rapidly at the higher pressing temperatures of 130–150 °C. The veneer stack heated the slowest at a pressing temperature of 100 °C. In this case, it took 60 s for the panel interior to reach 100 °C, which is equal to the temperature of the press plates (pressing temperature). By contrast, at 150 °C, the veneer stack was heated three times faster. These results are in good agreement with a previous study [4] that found that a considerable amount of thermal energy is needed to cure PF adhesives, i.e., the application of high temperatures (135–150 °C) or long pressing times (45–60 s/mm).

At low pressing temperatures of 100–120 °C, we observed gradual and slow heating of the panels. At the higher pressing temperatures of 130–150 °C, during the first 20 s, the panels heated very quickly before the moisture evaporated after which the heating rate slowed. Table 1 shows that the time required for the core layer of the three-layer panel to reach 100 °C was significantly reduced when the temperature was increased from 100 °C (60 s) to 120 °C (25 s) or 150 °C (19 s). After the interior temperature reached the temperature of water evaporation, the temperature inside the panel increased slowly until the core layer temperature was close to the pressing temperature.

Throughout the period of heat transfer between the sample and the hot press plate, the system remained at the veneer-heating stage. The heat was transmitted from the outside to inside, and the plywood gradually reached the pressing temperature, increasing from the surface layer to the core layer. At high pressing temperatures, such as 150 °C, for a short time (approximately 20 s), the core layer rapidly heated to 100 °C due to steam moving from the outer layers to the middle. However, during this time, the panel was densified, and its density increased while the porosity decreased, making it more difficult for the steam concentrated inside the panel to escape. If the steam cannot escape from the center of the panel, which is compressed between the press plates, then the steam will continue to increase the temperature of the center zone to the temperature of the press plates. A similar pattern of heating was previously described [4]. If the temperature increases from 20 to 100 °C, the conductivity slightly increases up to 14% and 24% in the longitudinal and transverse directions, respectively [31].

### 3.2. The Effect of Pressing Pressure on the Heating Rate of Non-Densified and Densified Veneer Stacks

Based on the results of the previous series of experiments, birch veneer and a plywood pressing temperature of 150 °C were used in subsequent studies.

**Figure 2:**
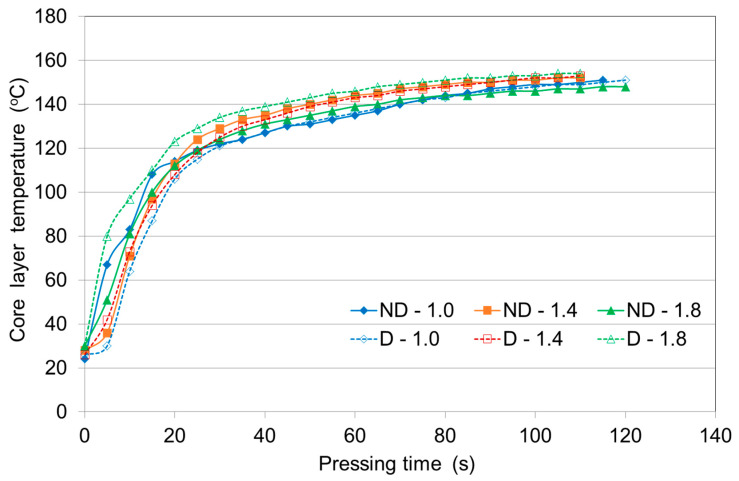
Core layer temperature curves of three-layer plywood made from non-densified (ND) or densified (D) birch veneers at different pressing pressures and 150 °C.

Figure 2 shows the dependence of the core layer temperature on the pressing pressure of the three-layer birch non-densified and densified veneer stacks. Table 2 shows the time required to reach a core layer temperature of 100 °C or a pressing temperature of 150 °C inside the three-layer panels when applying different pressures. There was practically no difference between densified and non-densified veneer stacks in the time required to reach an interior temperature of 100 °C when applying the different pressing pressures.

At pressures of 1.0, 1.4 and 1.8 MPa, a core layer temperature of 150 °C was reached after 110 s, 85 s and 125 s for panels made from non-densified veneers and after 115 s, 90 s and 75 s, for panels made from densified veneers, respectively. The data show that at the lower pressing pressures of 1.0 and 1.4 MPa, the panels heated at the same rate regardless of whether they were made of non-densified or densified veneer. The reason may be that panels have more porosity in the stack and make it relatively easy for steam to penetrate/migrate to the core while panels pressed at 1.8 MPa may mainly rely on heat conduction to raise the core temperature.

It is believed that the moisture content of the veneer stack affects the heat transfer from the outer layers to the panel core. Usually, higher moisture contents increase the thermal conductivity, which will accelerate the heat transfer. This may also be valid for low-density panels. The non-densified veneer had a higher moisture content (5.7%) than the densified veneer (1.4%). Arruda and Del Menezzi [32] also stated that thermomechanical treatment provided lower equilibrium moisture content of veneers. Therefore, for a panel made of non-densified veneer, we expected that its moisture content would have a significant effect on the rate of temperature rise. Data from Table 2 shows that densified veneer under high-pressure pressing was heated faster than the non-densified veneer at the same pressure. For the panel made of densified veneer, core temperatures of 100 °C and 150 °C were reached after 11 and 75 s, respectively, compared with 19 and 125 s for the non-densified veneer stack. Kurowska et al. [27] found that veneer densification shortened total pressing time by 12–25% in comparison to control samples. This phenomenon is caused by lower total moisture content in the veneer stack. Furthermore, faster stack internal temperature gain is caused by more dense wood substance. Wood compression mainly causes a reduction in empty spaces between cells and cell lumen [32]. Cai et al. [33] also showed that the panel moisture content does not have a significant effect on the time to reach the maximum core temperature.

The effective thermal conductivity of compressed wood was found to be lower than that of uncompressed wood [34]. On the other hand, Hrazsky and Kral [4] showed that the rate of heat passage increased with the increasing working pressure (within a certain pressing temperature range). However, in our study, this was only true for densified veneer. For non-densified veneers, it took longer to reach the pressing temperature inside the panel when applying high pressure. One of the reasons for this finding may be the higher moisture content of non-densified veneers compared with densified veneers. It is known that the rate of heat passage decreases with an increasing moisture content [4].

The most rapid heat transfer occurs when steam can pass through cracks and voids in the veneer. High pressures restrict these passages [20]. Wang et al. [30] similarly stated that during hot pressing the small deformations of the veneer ply effectively act as barriers to gas and moisture movement rather than the curing glue line acting in this regard. These barriers caused a sealing effect that led to a low rate of convection within the panel [30].

### 3.3. The Effect of the Number of Layers on the Heating Rate of Non-Densified and Densified Veneer Stacks

**Figure 3:**
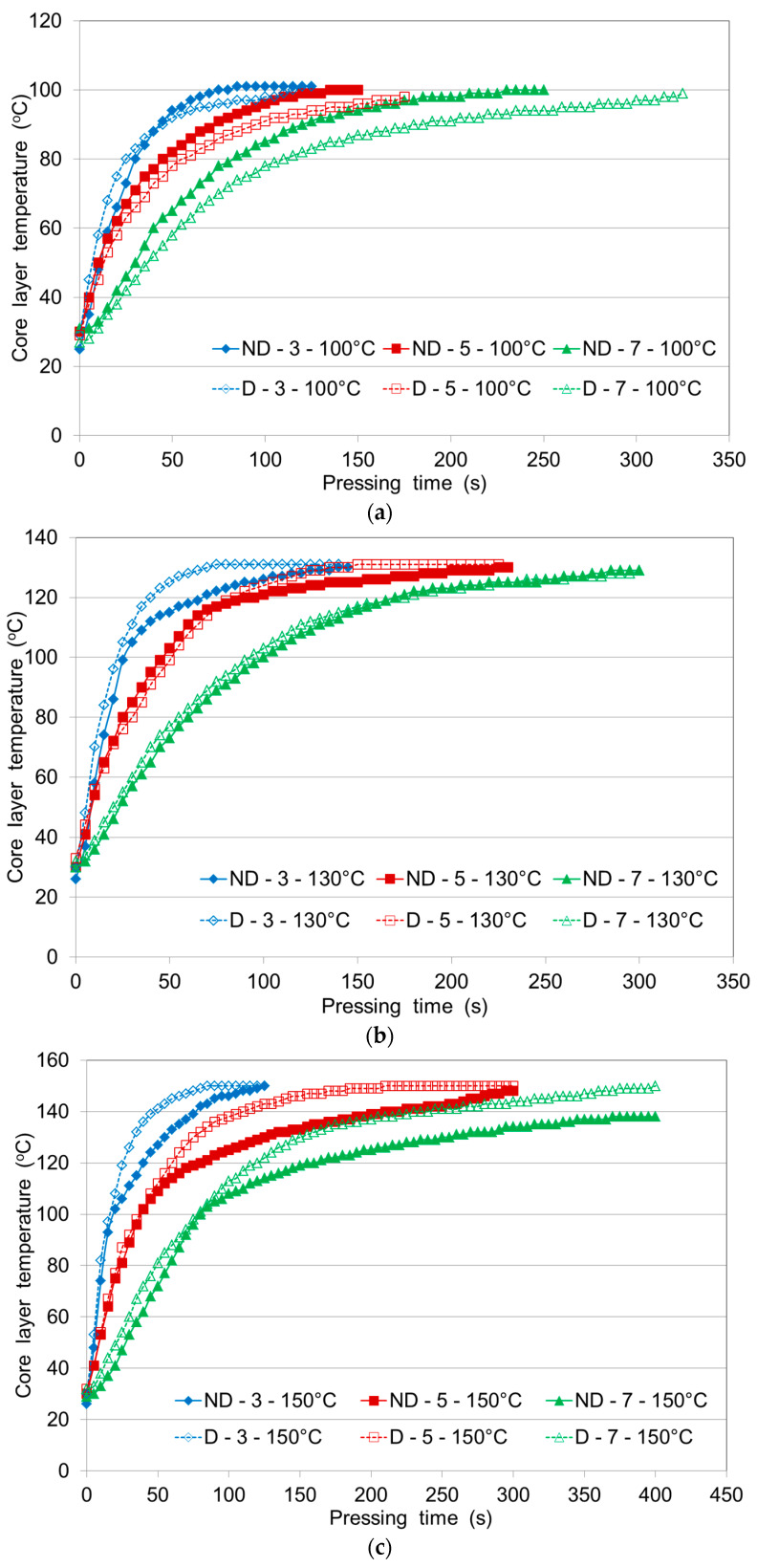
Core layer temperature curves of 3-, 5- and 7-layer plywood panels made from non-densified (ND) or densified (D) birch veneers at different pressing temperatures: (**a**) 100 °C; (**b**) 130 °C; (**c**) 150 °C.

Figure 3 shows the effect of the number of veneer layers (3, 5 and 7) on the heating rate of the non-densified and densified veneer stacks at different pressing temperatures (100 °C, 130 °C and 150 °C). The heating rate decreased significantly with an increased number of veneer layers, not only at the stage of rapid heating, but also at the stage of slow heating (Figure 3). With an increasing number of veneer layers, the time required for the core layer to reach 100 °C increased, as did the time required for the core layer to reach the pressing temperature (Figure 3).

With an increasing number of veneer layers, the temperature at which moisture evaporation occurred decreased with an increasing number of layers and the time period over which moisture evaporation occurred was prolonged, probably because the moisture evaporation required more heat due to the increased number of layers. The heat transfer rate decreased with the increase of veneer layers, so more time was required to reach 100 °C or the pressing temperature in the core layer, leading to an increasing time difference between reaching 100 °C and the pressing temperature (Figure 3). When there were too many veneer layers during hot pressing, the core layer temperature could not reach the pressing temperature even with an unlimited increase in pressing time, although the core layer temperature could approach the pressing temperature.

Figure 3a shows that the three-, five- and seven-layer panels of non-densified veneer heated faster at a pressing temperature of 100 °C than those of densified veneers. At the pressing temperature of 130 °C, the 3, 5 and 7-layer panels reached a core temperature of 100 °C in nearly the same time, regardless of densification. These results were also apparent at the pressing temperature of 150 °C. At pressing temperatures of 130–150 °C, for a further increase in core temperature above 100 °C, the densified veneer stacks heated to the pressing temperature faster than the non-densified veneer stacks and the difference between the pressing temperatures for the non-densified and densified veneers was already quite large. For example, the pressing temperature of 130 °C was reached in the core after 140, 225 and 325 s for the 3-, 5- and 7-layer non-densified veneers and after 70, 135 and 325 s for the densified veneers, respectively.

The pressing temperature of 150 °C was reached in the panel core after 125, 300 and 500 s for the 3-, 5- and 7-layer non-densified veneer stacks and after 85, 210 and 400 s for the 3-, 5- and 7-layer densified veneer stacks, respectively. Thus, the 3-, 5- and 7-layer panels of both the non-densified and densified veneers at pressing temperatures of 130 and 150 °C reached a core temperature of 100 °C in nearly the same time, but upon further heating, the densified panels heated much faster. The heating rate of the 3-, 5- and 7-layer panels to a temperature of 130 °C at a pressing temperature of 130 °C was faster by 50%, 40% and 0%, respectively, for the densified veneers compared with the non-densified veneers. The rate of heating to 150 °C at a pressing temperature of 150 °C for the 3-, 5- and 7-layer panels was faster by 32%, 30% and 20%, respectively, for the densified veneers compared with the non-densified veneers.

The heating rate decreased markedly with an increasing number of veneer layers. The effect of increasing the number of layers was mainly observed in the continuation of the first stage of constant temperature and the increase in time required for the core temperature to reach the pressing temperature. The heating rate of the core layer increased with increasing pressing temperature. In any case, a pressing temperature of 100 °C cannot be recommended for PF-bonded plywood panels for both technological and economic reasons. These findings are in good agreement with previous studies [4] who showed that temperatures of 135–150 °C or longer pressing times are required to cure PF adhesives.

Considering the curves (Figure 1, Figure 2 and Figure 3), we can conclude that the core temperature evolution can be divided into three stages as follows: in the first stage the core temperature remains constant for approximately 25–30 s after the platen reaches the veneer face surface; the second stage refers to the rapid increase in the core temperature due to convective heat flow; in the third stage the temperature remains nearly constant during the moisture vaporization. A similar core temperature distribution in the middle layers of multi-ply veneer assemblies of either non-densified or densified veneer stacks, as well as for when adhesives are not used, was previously observed [8]. The results of the temperature evolution inside the plywood samples for both densified and non-densified veneers were found to be quite similar to the heat transfer during hot pressing of particleboards and fiberboards [35,36].

### 3.4. The Influence of Pressing Pressure and Time on the Bonding Strength of Plywood Samples

**Figure 4:**
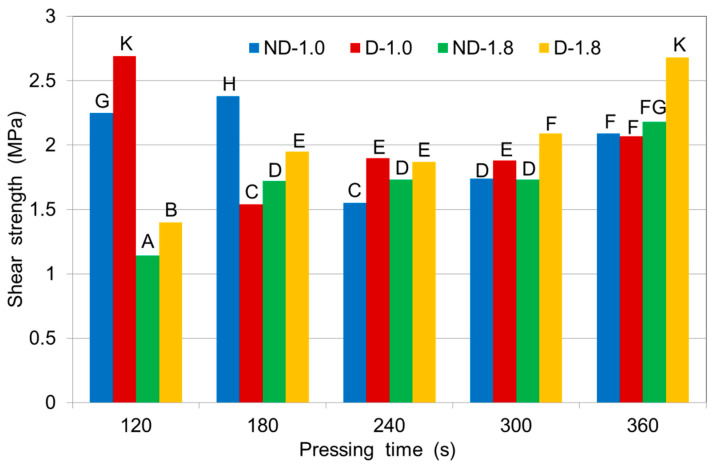
Shear strengths of 3-layer plywood samples made from birch veneers at various pressing pressures (1.0 and 1.8 MPa) for various durations at 150 °C and 100 g/m^2^ of adhesive spread: ND—non-densified veneer; D—densified veneer. Latin letters A–K indicate Duncan group.

The average shear strength values of the samples, along with the Duncan’s test results, are depicted in Figure 4. The shear strengths of the plywood samples composed of either densified or non-densified veneers were higher than 1.2 MPa and met the requirements of the EN 314-2 standard [29]. The highest shear strength was obtained by plywood samples composed of densified veneers made at a pressure of 1.4 MPa and a press times of 6 min. The lowest shear capacity was observed in samples composed of densified veneers made at a pressure 1.8 MPa using a 2 min press time. For densified veneers, the shear strength increased with increasing press time.

The shear strength values of the plywood samples made at a pressure of 1.8 MPa, but with different pressing times and at a lower adhesive spread rate (100 g/m^2^) were higher for the densified veneers than for the non-densified veneers (Figure 4). Increasing the pressing time of the samples from 120 to 360 s increased the shear strength and all shear strength values met the requirements of EN 314-2 [29]. The images of densified wood shows a significant improvement in the glue line, which became thinner and more continuous [32].

At a pressing pressure of 1.0 MPa and a lower adhesive spread rate (100 g/m^2^), the shear strengths of the plywood samples composed of densified veneers and made with pressing times of 120 s and 240–360 s were higher than shear strengths of the samples composed of non-densified veneers. In contrast, at a pressing time of 180 s, the shear strength values of the samples made from non-densified veneers were higher than of the samples from densified veneers (Figure 4).

For samples composed of densified veneers, the shear strength values for samples pressed at 1.0 MPa were lower than those made at 1.8 MPa. Even at 1.0 MPa, the shear strength values were high (>1.5 MPa) and met the requirements of EN 314-2 [29].

**Figure 5:**
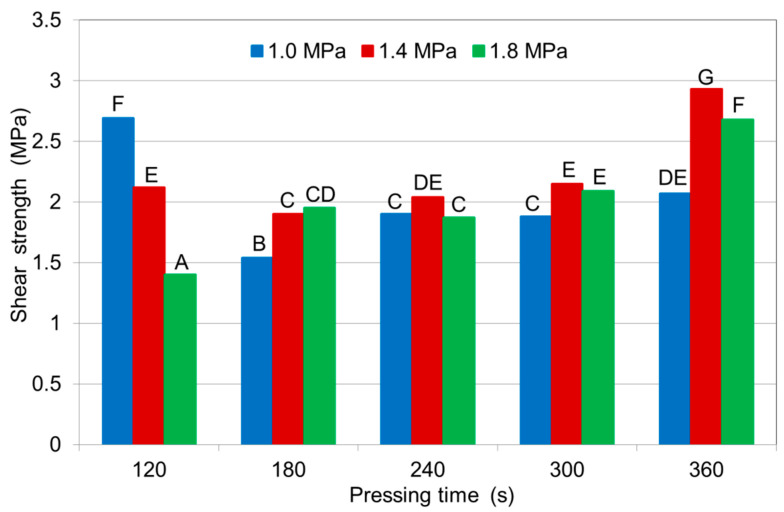
Shear strengths of 3-layer plywood samples made from densified birch veneers at various pressing pressures for various durations at 150 °C and 100 g/m^2^ of adhesive spread. Latin letters A–G indicate Duncan group.

For plywood samples made from densified veneers at a lower glue consumption rate (100 g/m^2^) and pressing times of 180–360 sec, higher pressures of 1.4 and 1.8 MPa led to higher shear strengths than when applying a pressure of 1.0 MPa (Figure 5). In contrast, for a pressing time of 120 s, the shear strength values of the samples at a pressure of 1.0 MPa were higher than the shear strengths of the samples made at 1.4 and 1.8 MPa.

Long pressing times at high pressing pressure can increase the veneer compression ratio and reduce the productivity of plywood, which is unacceptable in the plywood manufacturing industry. In terms of economics and technology, it is possible to choose a pressure of 1.4 MPa and a pressing time of 180–300 s. This allows the pressing time to be reduced by 17–50% and the pressing pressure by 22.2% without negatively impacting the bonding strength of the plywood. In similar study it was also found that densified veneers, except 25% pressing time shortening, allow 25% glue load reduction without affecting glue bonds strength properties [27].

**Figure 6:**
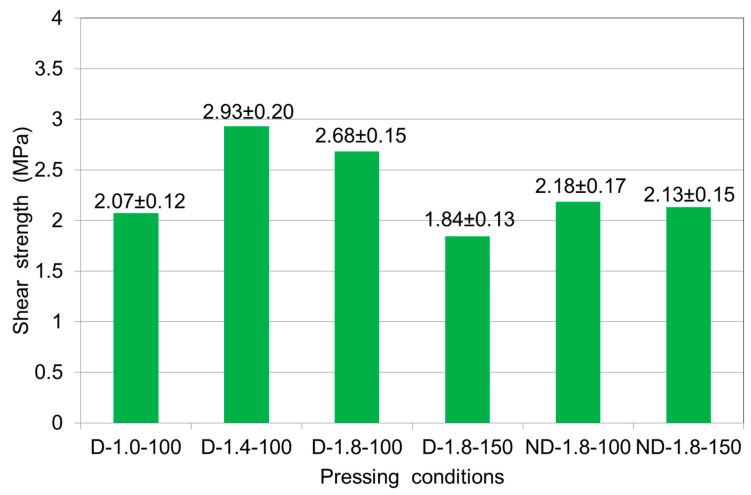
Shear strengths of 3-layer plywood samples made from birch veneers at various pressing pressures (1.0, 1.4 and 1.8 MPa) and a pressing time of 6 min for 150 and 100 g/m^2^ of adhesive spread: ND—non-densified veneer; D—densified veneer.

Figure 6 compares the bonding strengths of plywood samples made from either non-densified or densified veneers at reduced (100 g/m^2^) and currently accepted (150 g/m^2^) adhesive spread rates and at different pressing pressures. The highest bonding strength was observed for the densified veneers at a pressure of 1.4 MPa and the lower glue consumption of 100 g/m^2^. A lower bonding strength was found for samples made from densified veneer at a pressure of 1.8 MPa and an adhesive spread rate of 150 g/m^2^ than at a lower adhesive spread rate. Typically, the densification process smooths the surface of the veneer and decreases its roughness [37,38]; therefore, less adhesive is required for bonding. A lower adhesive consumption results in a reduced thickness of the adhesive layer and an increased bonding strength. An adhesive spread rate of 150 g/m^2^ for densified veneer is too large; the adhesive is squeezed out of the panel, the thickness of the adhesive layer increases and as a consequence, the adhesive strength decreases. It is known [39] that with increasing glue line thickness, the bonding strength decreases; with a thicker glue line, higher internal stress is generated during glue shrinkage, which can lead to a lower shear strength. Moreover, at the high glue spread level, gas pressure increased significantly due to the high MC in the glue line, which generally led to blisters or blows, which could largely deteriorate plywood bond quality [40].

At a pressing pressure of 1.4 MPa, the bonding strength was 2.93 MPa and was 8.5% higher than at a pressure of 1.8 MPa (2.68 MPa). At a pressing pressure of 1.8 MPa, but with an adhesive spread rate of 150 g/m^2^, the bonding strength (1.84 MPa) was 31.3% lower than with the lower adhesive spread rate (100 g/m^2^) at the same pressure. Wang et al. [40] also found that shear strength decreased as the pressing pressure increased. The high pressing pressure leads to reduced gas permeability and high internal gas pressure causing blisters or blows, which could completely destroy shear strengths of samples.

When comparing plywood samples made from densified and non-densified veneer made at a pressure of 1.8 MPa and an adhesive spread rate of 100 g/m^2^, the bonding strength of the densified samples was 18.7% higher (2.68 MPa) than of the non-densified samples (2.18 MPa). The opposite pattern was observed for the adhesive spread rate of 150 g/m^2^. The bonding strength of the non-densified samples was 13.6% higher (2.13 MPa) than for the densified samples (1.84 MPa). A previous study found that surface roughness will affect gluing and bonding between two layers of panels. It was observed that the adhesive was not distributed evenly on panels made from uncompressed veneer due to the effects of its rough surface in comparison with panels made from compressed veneer [41]. Non-densified veneer is rough and requires more glue, while densified veneer is smooth and has less roughness. The adhesive spread rate of 150 g/m^2^ was too large for the smooth veneer, which led to thickening of the adhesive layer and consequently reducing the bonding strength. Practice shows that the thicker the adhesive layer, the more noticeable the influence of internal stresses and, as a rule, the lower the bonding strength. For high-density hardwood veneers, a smooth surface is a necessity; where there is no surface contact, there can be no adhesion [14,42].

The veneer roughness plays an important role in the depth of penetration and the uniform distribution of the adhesive and influences the bonding quality of veneers. Arruda and Del Menezzi [37] also found that increasing the temperature or time led to a significant reduction in roughness. According to these authors, the veneer roughness decreased by 43.4%, which contributed to reducing the stress points between the veneer surface and the adhesive layer. Several studies [38,43,44,45,46] also determined that improved surface roughness of veneers increased the shear strength of the plywood made from them. Images of thermo-mechanically treated wood show a significant improvement in the glue line, which became thinner and more continuous [32]. Moreover, the permeability of the PF glue line decreased during glue curing and the permeability of cured glue lines (films) decreased with increasing glue spread [30]. The thermal conductivity of plywood increases with an increasing glue spreading rate by using phenol formaldehyde resin adhesive [47].

## 4. Conclusions

As a current contribution of the performed research, we can draw the following conclusions.

The heating rate of the veneer stacks increased as the pressing temperature increased. The panels were the slowest to heat at a pressing temperature of 100 °C, whereas at a pressing temperature of 150 °C, the panels heated to a core temperature of 100 °C three times faster. The heating rate of the core layer increased with increasing pressing temperature.

Practically, there was no difference in the time required to heat the core to a temperature of 100 °C for panels made of non-densified vs. densified veneers at the different pressing pressures. This pattern changed when heating the core to the pressing temperature. In this case, the densified veneer stacks heated faster than the non-densified panels at high pressing pressures. The heating rate of both the non-densified and densified veneer stacks decreased markedly with an increasing number of veneer layers. The 3-, 5- and 7-layer panels, for both the non-densified and densified veneers, reached a core temperature of 100 °C in nearly the same time at pressing temperatures of 130 and 150 °C. However, upon further heating, the densified veneer stacks heated much faster. The rate of heating to 150 °C for the 3-, 5- and 7-layer panels at a pressing temperature of 150 °C was faster by 32%, 30% and 20%, respectively, for the densified veneer than for the non-densified veneer.

When using densified veneers for the production of plywood, a shorter pressing time (17–50% reduction), lower glue consumption (33.3% reduction) and a lower pressing pressure (22.2% reduction) can be used without negatively impacting the bonding strength of the plywood samples.

The findings of this study provide useful information necessary for optimizing the plywood manufacturing process by balancing product qualities and productivity.

## Figures and Tables

**Table 1:** Time needed for the core layer of three-layer plywood bonded with PF adhesive to reach 100 °C and the pressing temperature.

Pressing Temperature (°C)	Time to Reach 100 °C (s)	Time to Reach the Pressing Temperature (s)
100	60	60
110	30	50
120	25	60
130	19	60
140	19	90
150	19	125

**Table 2:** Time needed for the core layer of three-layer plywood bonded with PF adhesive to reach 100 °C or the temperature of pressing.

Type of Veneer	Pressure of Pressing (MPa)	Time to Reach 100 °C (s)	Time to Reach 150 °C (s)
ND	1.0	14	110
ND	1.4	16	85
ND	1.8	19	125
D	1.0	18	115
D	1.4	17	90
D	1.8	11	75
